# Supplementary material for: The Influence of Different Pretreatment Methods on Color and Pigment Change in Beetroot Products
Source: Molecules. 2021 Jun 16;26(12):3683. doi: 10.3390/molecules26123683 (PMC8235720; doi:10.3390/molecules26123683)
Supplement: Supplementary file 1 [file molecules-26-03683-s001.zip › molecules-1235474-SI.pdf]

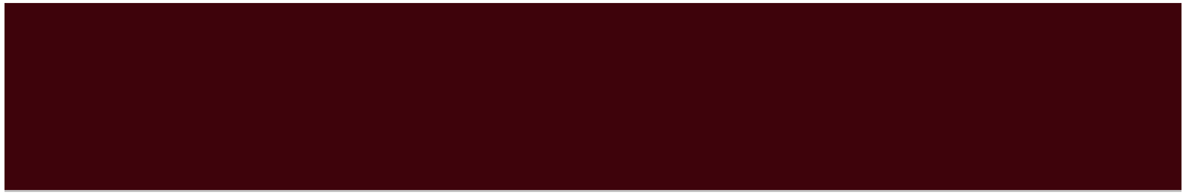

Figure S1. Color of beetroot obtained from color measurement

Pre-treatment	Juice obtained after FD		
-	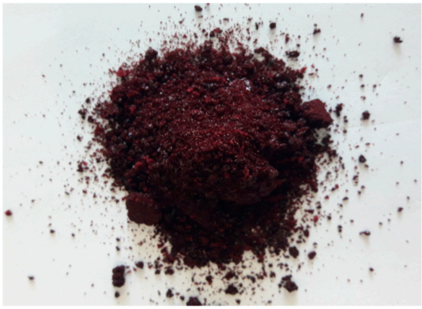		
Steam 10 min	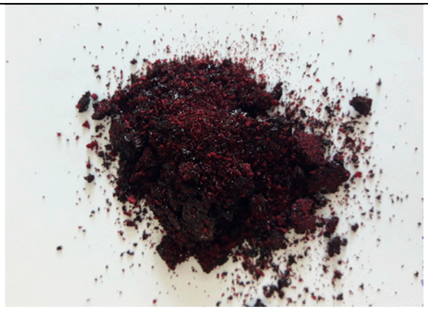	Steam 15 min	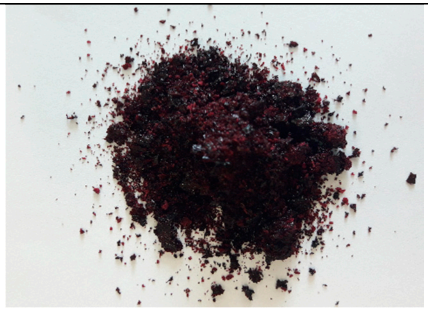
US 10 min	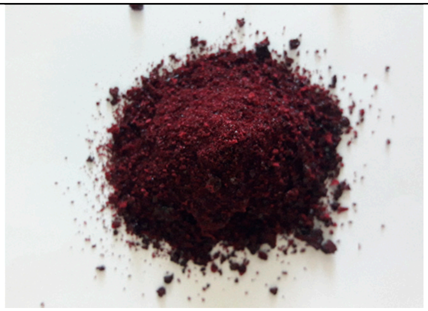	US 15 min	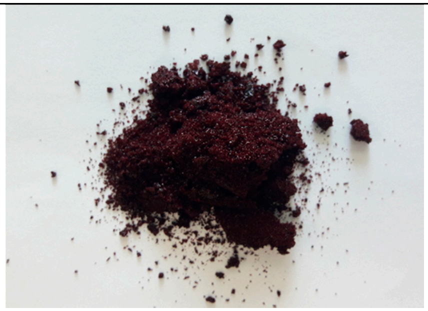

Figure S2. Outer morphology of beetroot powders obtained after Freeze-Drying (FD),

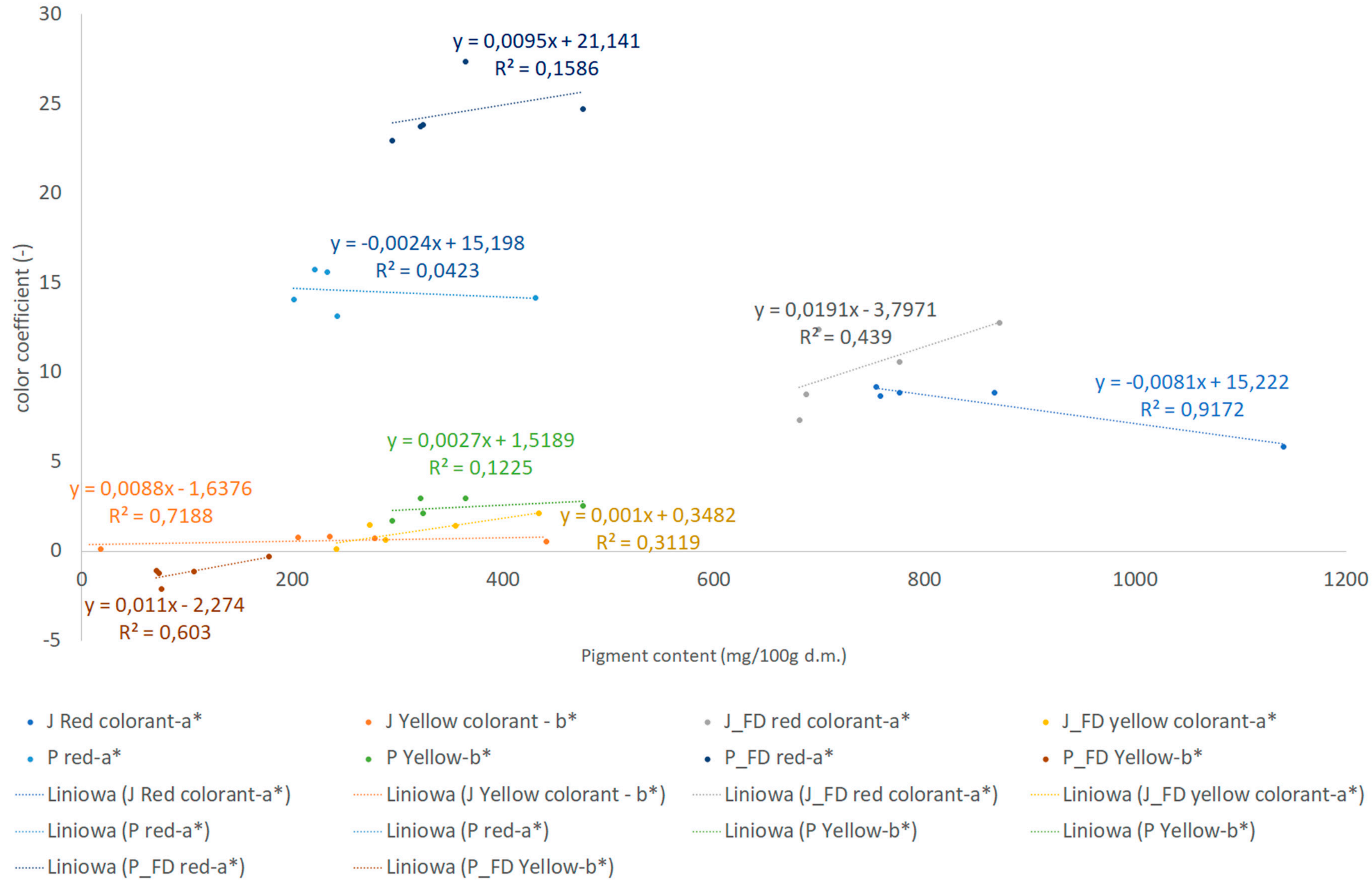

Figure. S3. Correlation between the colorant content (Red-betalain, Yellow- vulgaxanthin-I) and the color coefficient a* or b*
